# Detection of Activities by Wireless Sensors for Daily Life Surveillance: Eating and Drinking

**DOI:** 10.3390/s90301499

**Published:** 2009-03-03

**Authors:** Sen Zhang, Marcelo H Ang, Wendong Xiao, Chen Khong Tham

**Affiliations:** 1 Department of Electrical and Computer Engineering, National University of Singapore, 4 Engineering Drive 3, Singapore 117576; 2 Department of Mechanical Engineering, National University of Singapore, 9 Engineering Drive 1, Singapore 117576; 3 Networking Protocols Department, Institute for Infocomm Research, 1 Fusionopolis Way, No. 21-01 Connexis, South Tower, Singapore 138632

**Keywords:** Wireless Sensor, HTM, Feature Extraction, Eating and Drinking, Euler Angle

## Abstract

This paper introduces a two-stage approach to the detection of people eating and/or drinking for the purposes of surveillance of daily life. With the sole use of wearable accelerometer sensor attached to somebody’s (man or a woman) wrists, this two-stage approach consists of feature extraction followed by classification. At the first stage, based on the limb’s three dimensional kinematics movement model and the Extended Kalman Filter (EKF), the realtime arm movement features described by Euler angles are extracted from the raw accelerometer measurement data. In the latter stage, the Hierarchical Temporal Memory (HTM) network is adopted to classify the extracted features of the eating/drinking activities based on the space and time varying property of the features, by making use of the powerful modelling capability of HTM network on dynamic signals which is varying with both space and time. The proposed approach is tested through the real eating and drinking activities using the three dimensional accelerometers. Experimental results show that the EKF and HTM based two-stage approach can perform the activity detection successfully with very high accuracy.

## 1. Introduction

Tracking and identification of daily physical activities are key factors to evaluate the quality of life and health status of a person. Research on this field is well recognized in rehabilitation, assessment of physical treatment [1, 2] and is shown to have significant impacts on the health care of elderly persons and patients [3]. For example, Great Eastern Life Insurance Company has defined the elder people’s disability as: the inability of the Policyholder to perform at least 3 Activities of Daily Living (washing, dressing, feeding, toileting, mobility and transferring), even with the aid of special equipments, and always to require the physical assistance of another person throughout the entire activity. In these activities, feeding means the ability to feed oneself food after it has been prepared and made available. Therefore, eating and drinking detection is a very important topic for daily life surveillance. Measurement of eating or drinking activities in daily life or continuous recording of these activities at home would provide more reliable diagnosis of disabilities for hospitals or insurance companies. However, eating and drinking detection poses a challenge for the state of the art of the research in activity recognition [4], and few references or systematic methods can be found in the literature.

In the daily life surveillance system, if the human activities (such as eating or drinking) can be tracked accurately, the results can help greatly and readily improve the ability of the identification of the whole system. Therefore, devices that can accurately track the pose of limbs in space are essential components of such a surveillance system.

One method of tracking and monitoring activities is via tracking the pose of human limbs in space. The human limb tracking system can be classified as non-vision based and vision-based systems. Non-vision based systems use inertial, mechanical and magnetic sensors *etc.* to continuously collect movement signals. For example, the Micro-ElectroMechanical Systems (MEMS) inertial and magnetic sensor devices [5, 6, 7, 8] can be used in most circumstances without limitations (i.e. illumination, temperature, or space, *etc.*) and show better performance in accuracy against mechanical sensors. The main drawback of using inertial sensors is that accumulating errors (or drift) can become significant after a short period of time. Vision-based systems are widely used in recent years, such as [9, 10, 11, 12]. However, most vision-based approaches to human movement tracking involve intensive computations, such as temporal differencing, background subtraction or occlusion handling. In many cases, once a prior knowledge of an estimation of object kinematics is available, the expensive image detector array appears inefficient and unnecessary.

Accelerometry-based activity analysis has been developed fast in recent years. Some prototype systems which aim at monitoring daily activities [13], conducting gait analysis [14], *etc.* are reported. In our system, the 3D accelerometers are applied to collect raw measurement data of the moving arm and the server computer communicates with the sensor devices via the blue-tooth. The simple hardware structure makes the data acquisition and processing easy. In this paper, a combined two-stage recognition approach is proposed for the eating and drinking detection for the daily life surveillance. A kinematics model of human forearm movements in three dimension is developed and the Extended Kalman Filter (EKF) is applied to extract features from the 3D accelerometer signals (raw data). This will greatly improve the recognition results compared to using the raw data as the inputs of the Hierarchical Temporal Memory (HTM) network. After the feature extraction, the HTM algorithm is applied for the recognition purpose. HTM has the advantage that it can classify the dynamic signals which vary with both time and space due to its hierarchical memory and the belief propagation mechanisms.

To the best of our knowledge, no work can be found for eating and drinking activity detection based on feature extraction algorithms. Our main contribution is the novelty of the two-stage approach and feature extraction applied to the eating/drinking detection. This method not only improves the accuracy of the activity detection compared to using the raw data, but also provides the basis for the time and space varying activities’ identification by using HTM algorithm.

The layout of the paper is as follows: Section 2 presents the related work to arm gesture classifications. Section 3 describes the system hardware and the wireless accelerometer we used in this paper. Section 4 proposes feature extraction algorithm we derived. Section 5 describes how the HTM works and proposes our own design using HTM network for eating/drinking detection. Section 6 reports the simulation and experimental results. Conclusions and future work are given in Section 7.

## 2. Related Work

The following text describes relevant work that utilizes human model-based approaches involving hand and arm movements and gestures. The comparison between the HTM algorithm and the relevant work is also presented.

The common methodologies that have been used for arm gesture recognition are: (1) template matching [15]; (2) neural networks [15]; (3) statistical method, and (4) multi-modal probabilistic combination [16]. The template approach compares the unclassified input sequence with a set of predefined template patterns. The algorithm requires preliminary work for generating the set of gesture patterns, and has poor recognition performance typically due to the difficulty of aligning the input with the template patterns [19].

By far the most popular recognition methods are the neural networks (e.g., [17]) and the statistical method–Hidden Markov Models (HMMs) (e.g., [18]).

The Neural Network (NN) approach works by pre-determining a set of common discriminating features, estimating covariances during a training process, and using a discriminator to classify gestures. The drawback of this method is that features are manually selected and time consuming training is involved [15]. The NN does not exploit temporal coherence between the features as HTM do.

The HMMs method is a variant of a finite state machine characterized by a set of states, a set of observation symbols for each state, and probability distributions for state transitions, observation symbols and initial states [20]. The state transitions, which are hidden to the observer, generate an observation symbol from each state. The basic premise of the HMMs is to infer a state sequence that produces a sequence of observations. Learning the state sequence can help to understand the structure of the underlying model that generates the observation sequence. The major drawbacks of the HMMs are: (1) they require a set of training gestures to generate the state transition network and tune parameters; (2) they make assumptions that successive observed operations are independent, which is typically not the case with human motion and speech [20].

In the statistical methods, Hierarchical Hidden Markov Model (HHMMs) [21] and Bayesian networks [22] come closest to the way HTM model time, modelling the nested structure of time in a hierarchy. However, the hierarchy that is exploited in HHMMs is only in one dimension (usually time). HTM has a hierarchy in space and time. This gives HTM several unique advantages while learning about the world. Moreover, the theory of HTM includes provisions for using activities and attention to learn the world.

Support Vector Machine (SVM) [23, 24] is an efficient way to find boundaries in a high dimensional space that separate the various examples into their labelled categories. It does not make any assumptions about the hierarchical or temporal organization of the world and hence cannot exploit these properties for efficient learning. Since the underlying model of SVM is discriminative and not generative, it cannot be used to predict forward in time.

HTM uses a unique combination of the following ideas [24]: 1) A hierarchy in space and time to share and transfer learning; 2) Slowness of time, which, combined with the hierarchy; enables efficient learning of intermediate levels of the hierarchy; 3) Learning of causes by using time continuity and actions; 4) Models of attention and specific memories; 5) A probabilistic model specified in terms of relations between a hierarchy of causes; 6) Belief propagation in the hierarchy to use temporal and spatial context for inference.

From the above analysis of different approaches, we can see that the HTM method has the advantages as follows: it can classify the dynamic signals which are variable with both time and space because of the hierarchical memory and the belief propagation. Based on the features extracted by EKF, the HTM can greatly improve the accuracy of the activity detection. Compared to the different traditional methods mentioned above, it is a promising research tool in the activity detection and classification area.

## 3. System Hardware

**Figure 1.**
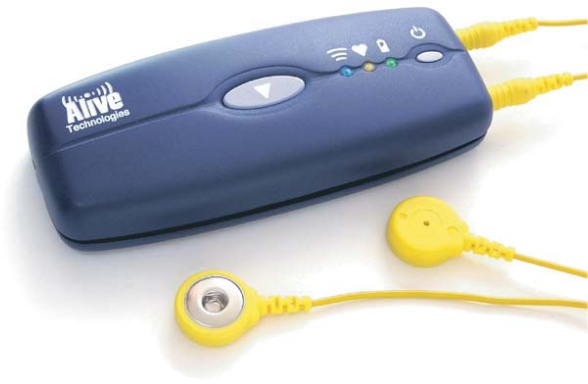
The sensor in our experiment

In our system, the product of Alive Technologies named Mobile Cardiac Monitor is applied, see Figure 1. It is a wireless health monitoring product for screening, diagnosis and management of chronic diseases, and for consumer health and fitness. Applications include the management of atrial fibrillation and heart failure, cardiac rehabilitation and fitness monitoring. Designed for use in the doctor’s office, home or gym, the monitor uses wireless blue-tooth and mobile phone networks to immediately transmit accelerometer data or other data such as heart rate to a computer, PDA, or central monitoring center. Although it combines several sensors’ functions, the 3-axis accelerometer inside the monitor is our concern. This device and one computer constitute our eating and drinking detection system.

## 4. Euler Angle Tracking for Arm Movement

In this work, we attempt to recognize successive arm movements of the manoeuvring sub-phase to and from the mouth using the inertial sensor–accelerometer. In order to identify the arm movement, the features have to be extracted. Here, we use Euler angles *α*, *β*, *γ* to describe rotations or relative orientations of the arm. The angles *α*, *β*, *γ* describe successive rotations about the fixed *x*, *y*, *z* axes [25]. We consider the arm moving in a 3-D Cartesian coordinate system that we formulate in the next section. The system states which represent the arm movement features include the arm angular velocity and the arm Euler angles.

### 4.1. The Arm Movement Model

**Figure 2.**
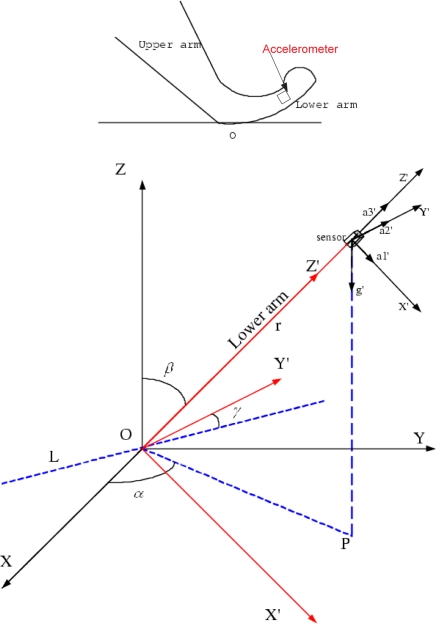
The 3-D arm movement system.

Consider a rigid human forearm moving in the 3-D space. Figure 2 shows the kinematics of the human lower arm, where the elbow is fixed at *o* and the accelerometer is attached near the wrist. *r* is the distance between the center of the sensor and *o* that is defined as the system origin. The figure also shows the relationship between the reference coordinate system and the sensor coordinate system. *X – Y – Z* denotes the reference Cartesian coordinate system and *X*′ *– Y*′ *– Z*′ is the sensor frame. Readings of accelerometer are along the axis of the frame. Here, we choose the table for eating/drinking as the *X – Y* plane of the reference coordinate system and the origin is chosen as the elbow of the person who is eating. Thus *Z* axis is automatically fixed. In Figure 2, dotted line *L* is the intersection line between the plane *X – Y* of the reference coordinate system and the plane *X*′ *– Y*′ of the sensor coordinate system. Thus the Euler angle for the arm movement system is *α*, *β*, *γ* according to the definition in [25].

In the sensor coordinate system, the accelerometer’s readings are *a*_1′_, *a*_2′_, *a*_3′_ in three axis directions. The gravity is *g*′ in the sensor coordinate system. Assume that the Euler angles at time step *k* are *α*(*k*), *β*(*k*), *γ*(*k*) in the reference coordinate system. The angular velocities are *α̇*(*k*), *β̇*(*k*), *γ̇*(*k*) and the sampling time interval is *T*. We assume that during the sampling time interval, the angular velocity is a constant. This is a reasonable approximation if sampled period is small. We can write the system state equations as follows:

(1) x(k+1)=F(k)x(k)+v(k),

where **x**(*k* + 1) = [*α*(*k* + 1) *α̇*(*k* + 1) *β*(*k* + 1) *β̇*(*k* + 1) *γ*(*k* + 1) *γ̇*(*k* + 1)]*^T^*

$$\begin{array}{l} F(k)=\left[\begin{array}{llllll} 1 & T & 0 & 0 & 0 & 0\\ 0 & 1 & 0 & 0 & 0 & 0\\ 0 & 0 & 1 & T & 0 & 0\\ 0 & 0 & 0 & 1 & 0 & 0\\ 0 & 0 & 0 & 0 & 1 & T\\ 0 & 0 & 0 & 0 & 0 & 1 \end{array}\right],\\ v(k)={\left[\begin{array}{llllll} v_{\alpha } & v_{\dot{\alpha }} & v_{\beta } & v_{\dot{\beta }} & v_{\gamma } & v_{\dot{\gamma }} \end{array}\right]}^{T}. \end{array}$$

*v_α_*, *v_α̇_*, *v_β_*, *v_β̇_*, *v_γ_*, *v_γ̇_* are noise of the respective state variables. They are assumed to be independent, zero mean Gaussian noise with distribution function: *P*(*v_α_*, *v_α̇_*, *v_β_*, *v_β̇_*, *v_γ_*, *v_γ̇_*) ∼ *N*(0, **Q**(*k*)),where

$$Q(k)=\left[\begin{array}{cccccc} Q_{\alpha } & 0 & 0 & 0 & 0 & 0\\ 0 & Q_{\dot{\alpha }} & 0 & 0 & 0 & 0\\ 0 & 0 & Q_{\beta } & 0 & 0 & 0\\ 0 & 0 & 0 & Q_{\dot{\beta }} & 0 & 0\\ 0 & 0 & 0 & 0 & Q_{\gamma } & 0\\ 0 & 0 & 0 & 0 & 0 & Q_{\dot{\gamma }} \end{array}\right]$$

is the covariance matrix and *Q_α_*, *Q_α̇_*, *Q_β_*, *Q_β̇_*, *Q_γ_*, *Q_γ̇_* are variances of the respective variables.

### 4.2. System Observation Model

In order to build up the estimation scheme, the sensor observation model is needed. In the sensor Cartesian coordinate system, the sum of the accelerations should be zero assuming that the arm moves with the constant velocity for each sampled period *kT* ≤ *t* ≤ (*k* + 1)*T*. The assumption of zero accelerations is reasonable if the sampling time is very small because of the velocity of the arm is almost unchanged during such a small period. Thus, we have:

(2) $$g^{\prime }(k)=\sum_{i=1}^{3}a_{i}^{\prime }(k)=\left[\begin{array}{c} a_{1}^{\prime }(k)\\ a_{2}^{\prime }(k)\\ a_{3}^{\prime }(k) \end{array}\right].$$

where the **g**′(*k*) and **a**′*_i_*(*k*) are vectors and *a*′*_i_*(*k*) is the reading from the accelerometer at time step *k*. According to the coordinate transformation relationship in [25], we have:

(3) $$g^{\prime }(k)=R_{\mathrm{xyz}}\;\;\left[\begin{array}{c} 0\\ 0\\ -g \end{array}\right],$$

where **R***_xyz_* is the transformation matrix from the fixed reference frame to sensor frame, and *g* is the gravity in the reference coordinate system. According to [25], we know:

(4) $$R_{\mathrm{xyz}}=\left[\begin{array}{ccc} R_{1} & R_{2} & R_{5}\\ R_{3} & R_{4} & R_{6}\\ R_{7} & R_{8} & \text{cos}\;\beta (k) \end{array}\right],$$

where

$$\begin{array}{c} R_{1}=\text{cos}\;\alpha (k)\;\text{cos}\;\beta (k)\;\text{cos}\;\gamma (k)-\text{sin}\;\alpha (k)\;\text{sin}\;\gamma (k),\\ R_{2}=\text{sin}\;\alpha (k)\;\text{cos}\;\beta (k)\;\text{cos}\;\gamma (k)+\text{cos}\;\alpha (k)\;\text{sin}\;\gamma (k),\\ R_{3}=-\text{cos}\;\alpha (k)\;\text{cos}\;\beta (k)\;\text{sin}\;\gamma (k)-\text{sin}\;\alpha (k)\;\text{cos}\;\gamma (k),\\ R_{4}=-\text{sin}\;\alpha (k)\;\text{cos}\;\beta (k)\;\text{sin}\;\gamma (k)+\text{cos}\;\alpha (k)\;\text{cos}\;\gamma (k),\\ R_{5}=-\text{sin}\;\beta (k)\;\text{cos}\;\gamma (k),\\ R_{6}=\text{sin}\;\beta (k)\;\text{cos}\;\gamma (k),\\ R_{7}=\text{cos}\;\alpha (k)\;\text{sin}\;\beta (k),\\ R_{8}=\text{sin}\;\alpha (k)\;\text{sin}\;\beta (k), \end{array}$$

From equations (2) and (3), we can write the measurement model as:

(5) $$\left[\begin{array}{c} a_{1}^{\prime }(k)\\ a_{2}^{\prime }(k)\\ a_{3}^{\prime }(k) \end{array}\right]=R_{\mathrm{xyz}}\;\;\left[\begin{array}{c} 0\\ 0\\ -g \end{array}\right]+w(k),$$

i.e.

(6) $$\{\begin{array}{l} a_{1}^{\prime }(k)=\text{sin}\;\beta (k)\;\text{cos}\;\gamma (k)\cdot g+w_{1}(k)\\ a_{2}^{\prime }(k)=-\text{sin}\;\beta (k)\;\text{sin}\;\gamma (k)\cdot g+w_{2}(k)\\ a_{3}^{\prime }(k)=-\text{cos}\;\beta (k)\cdot g+w_{3}(k). \end{array}$$

Or in matrix form,

(7) Z(k)=h(x(k))+w(k),

where *h* is a non-linear measurement function depending on sensor’s measurement characteristic. **w**(*k*) is a variable representing measurement noise in sensor. It is assumed to be zero-mean Gaussian distribution white noise. The covariance of **w**(*k*) is *R*(*k*).

The measurement model is a non-linear function, for the estimation purpose, the linearization is needed. Thus, we have to calculate the Jacobian matrix as follows:

$$H(x(k))=\left[\begin{array}{cccc} \text{cos}\;\beta \;\text{cos}\;\gamma \cdot g & 0 & -\text{sin}\;\beta \;\text{sin}\;\gamma \cdot g & 0\\ -\text{cos}\;\beta \;\text{sin}\;\gamma \cdot g & 0 & -\text{sin}\;\beta \;\text{cos}\;\gamma \cdot g & 0\\ \text{sin}\;\beta \cdot g & 0 & 0 & 0 \end{array}\right].$$

From (6), it can be seen that *α* does not affect the accelerometer’s readings. Ignore the first two rows of (1) (*α*, *α̇* are not observable), we can obtain the following system model for the estimation of the arm movement:

(8) $$\left[\begin{array}{c} \beta (k+1)\\ \dot{\beta }(k+1)\\ \gamma (k+1)\\ \dot{\gamma }(k+1) \end{array}\right]=\left[\begin{array}{cccc} 1 & T & 0 & 0\\ 0 & 1 & 0 & 0\\ 0 & 0 & 1 & T\\ 0 & 0 & 0 & 1 \end{array}\right]\;\;\;\left[\begin{array}{c} \beta (k)\\ \dot{\beta }(k)\\ \gamma (k)\\ \dot{\gamma }(k) \end{array}\right]+v(k).$$

Based on the above system model and observation model, the EKF can be used to estimate the system variables.

### 4.3. Extended Kalman Filter for Estimation

Based on the above system model, an extended Kalman filter [26] is used to implement the state prediction and update.

Assume the system equation is as Equation (1). Given the estimate **x̂**(*k* | *k*) of **x**(*k*), the predicted state **x̂**(*k* + 1|*k*) using (1) is given by

(9) $$\hat{x}(k+1|k)=F(k)\;(\hat{x}(k|k)).$$

The prediction error covariance matrix is approximated by:

(10) $$P(k+1|k)=F(k)P(k|k)F^{T}(k)+Q(k).$$

In view of the system observation model:

(11) Z(k)=h(x(k))+w(k),

the predicted measurement is simply:

(12) $$\hat{Z}(k+1)=H(x(k))\hat{x}(k+1|k).$$

Then, the difference between the measurement and the predicted observation, named the innovation, is given by:

(13) $$\nu (k+1)=Z(k+1)-\hat{Z}(k+1)$$

(14) $$=h(x(k))-H(x(k))\hat{x}(k+1|k)+w(k).$$

Thus, the covariance of the innovation is:

(15) $$s(k+1)=H(x(k))P(k+1|k)H{\left(x(k)\right)}^{T}+\sigma_{r}^{2}.$$

The EKF gain is given by:

(16) $$K(k+1)=P(k+1|k)H{\left(x(k)\right)}^{T}s^{-1}(k+1).$$

We update the estimation using the following equations:

(17) $$\hat{x}(k+1|k+1)=\hat{x}(k+1|k)+K(k+1)\nu (k+1),$$

(18) $$P(k+1|k+1)=P(k+1|k)-K(k+1)s(k+1)K^{T}(k+1).$$

## 5. Hierarchical Temporal Memory Algorithm

### 5.1. The HTM Structure and How it Works

**Figure 3.**
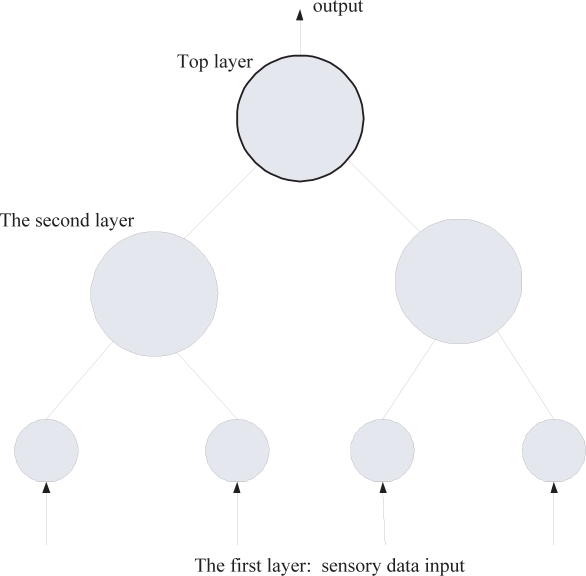
A simple HTM network structure.

A HTM is structured as a hierarchy of nodes, where each node is performing the same learning algorithm. Figure 3 shows a simple HTM hierarchy. Measurement data from sensors (sensory data) enters at the bottom. Exiting the top is a vector where each element of the vector represents a potential cause of the sensory data. Potential cause means the possible objects that give the sensory data. Each node in the hierarchy performs the same function as the overall hierarchy. That is, each node looks at the spatial-temporal pattern of its input and learns to assign causes to this input pattern. Spoken simply, each node, no matter where it is in the hierarchy, discovers the causes of its inputs. The outputs of nodes at one level become the inputs to the next level in the hierarchy. Nodes at the bottom of the hierarchy receive input from a small area of the sensory input. Therefore, the causes that they discover are the ones that are relevant to a small part of the sensory input area. Higher up regions receive input from multiple nodes below, and again discover the causes in this input. These causes will be of intermediate complexity, occurring over larger areas of the entire input space. The node or nodes at the top of the hierarchy represent high level causes that may appear anywhere in the entire sensory field. For example, in a visual inference HTM, nodes at the bottom of the hierarchy will typically discover simple causes such as edges, lines, and corners in a small part of the visual space. Nodes at the top of the hierarchy will represent complex causes such as dogs, faces, and cars which can appear over the entire visual space or any sub-part of the visual space. Nodes at intermediate levels in the hierarchy represent causes of intermediate complexity that occur over intermediate-sized areas of the visual space.

### 5.2. Learning Algorithm in One Node

The HTM nodes consist of the following components: 1) A spatial pooler, which finds meaningful coincidences in its inputs. 2) A temporal pooler which groups coincidences that occur nearby in time. 3) supervised mapper (for supervised learning) which associates coincidences with categories received from a category sensor. The spatial pooler, temporal pooler, and supervised mapper are the key substructures of the nodes that perform learning and inference [27].

During the learning mode, the spatial pooler analyzes the stream of input vectors in order to generate a coincidence matrix. This coincidence matrix quantizes the potentially huge space of all possible input vectors into a relatively small, finite set of representative canonical inputs. The algorithm applied in this phase is Maxdistance: if the distance between two vectors is smaller than a defined Maxdistance, they will be thought the same group, i.e., if the squared distance between an input vector *x* and an existing coincidence *w* is less than Maxdistance, the input vector is not considered novel and is (conceptually) pooled together with that existing coincidence; the details of the algorithm can be found in [27]. The coincidence matrix starts out empty. When the spatial pooler selects a particular input vector to be a coincidence, it simply appends this input vector to the coincidence matrix as a new row. For example, there are 6 vectors: *x*_1_, *x*_2_, *x*_3_, *x*_4_, *x*_5_ and *x*_6_, let *W* denotes the coincidence matrix. In the initialization, *W* = [*x*_1_]; If the distance between the second coming vector *x*_2_ and *x*_1_ is less than Maxdistance, *W* will be unchanged; Otherwise, *W* will be changed to be [*x*_1_; *x*_2_]. Until the 6 vectors are all processed, *W* is then formed.

Once the node is switched to inference mode, the spatial pooler no longer updates the coincidence matrix, and instead compares each new input vector to the coincidences in the coincidence matrix. During inference, the spatial pooler computes a belief vector *y* for its input vector *x*. This output vector is a distribution over coincidences, so it contains one element for each row in the coincidence matrix. The spatial pooler computes the belief according to the equation for the *j*th coincidence: *y_j_* = *e*^−||*x–W_j_*||^2^/2*σ*^2^^; For example [27], during learning mode, the pooler generated a coincidence matrix W containing the following three coincidences:

$$W=\left[\begin{array}{cccccccc} 1 & 2 & 2 & 6 & 1 & 4 & 5 & 8\\ 9 & 8 & 9 & 1 & 0 & 2 & 1 & 4\\ 5 & 5 & 4 & 6 & 5 & 4 & 6 & 6 \end{array}\right].$$

Assuming that the pooler receives the following input vector during inference:

$$x_{1}=\left[\begin{array}{cccccccc} 3 & 3 & 4 & 5 & 2 & 4 & 6 & 9 \end{array}\right].$$

The input vector *x*_1_ is presented, and the pooler computes the squared distances to each of the three coincidence vectors stored in *W* as 13, 160, and 27, respectively. The pooler converts these squared distances into belief values using the Gaussian model:

$$\begin{array}{c} y_{1}=e^{-13/2\sigma^{2}}=0.771,\\ y_{2}=e^{-160/2\sigma^{2}}=0.041,\\ y_{3}=e^{-27/2\sigma^{2}}=0.583. \end{array}$$

Here we assume that the node has been configured with *σ* equal to 5.0, the square root of Maxdistance. These three belief values are assembled into the spatial pooler output belief vector *y*_1_:

$$y_{1}=\left[\begin{array}{ccc} 0.771 & 0.041 & 0.583 \end{array}\right].$$

The detailed algorithm (Gaussian Inference/Dot Inference)and equations can be seen in [27]. In this context, the term belief represents a generalized measure of the likelihood that a particular input vector *x* and a particular coincidence *w* both represent the same underlying real-world cause.

The output vector *y* is handed off to the temporal pooler. In fact, the spatial pooler can be thought of as a pre-processor for the temporal pooler. It simplifies the temporal pooler task by pooling the vast space of input vectors into a relatively small set of discrete coincidences that are easier to handle. The coincidence matrix and the corresponding output vector is the input of the temporal pooler. The job of the temporal pooler is to group together temporally-related coincidences. During learning, the temporal pooler receives coincidence indices sent by the spatial pooler, and it keeps track of which ones occurred close together in time. The temporal pooler builds the time-adjacency matrix, which keeps track of transitions between coincidences. After learning is completed, the pooler forms non-overlapping groups of coincidences, with each group containing coincidences that often followed each other during learning. For each of past coincidences, the pooler increments the value in the time-adjacency matrix corresponding to a transition from the past coincidence to the current coincidence. Thus the time-adjacency matrix can express the times that one coincidence occurred during the past learning process. During inference, the temporal pooler builds a list of groups from the time-adjacency matrix. It also creates a matrix of weights, using the coincidence frequency counts maintained by the spatial pooler. The temporal pooler uses its list of groups to convert incoming belief vectors to distributions over groups. The algorithm (maxProp) for this phase can be found in [27]. For each group, the maxProp algorithm finds the coincidence in that group with the highest value in the belief vector received from the spatial pooler. That maximal belief in the group becomes the value for the group itself, and it is entered into the output vector. To illustrate [27], let the input *y* from the spatial pooler be as follows, representing beliefs over five coincidences:

$$y=\left[\begin{array}{ccccc} 0.04 & 0.12 & 0.30 & 0.01 & 0.22 \end{array}\right].$$

And let the groups be: group 0 contains coincidences 1, 3, and 4, and group 1 contains coincidences 0 and 2. The output of the temporal pooler *z* is the following:

$$z=\left[\begin{array}{cc} 0.22 & 0.30 \end{array}\right].$$

Here, *z* contains the highest value in *y* for all coincidences in group 0 (0.22, from coincidence 4), as well as the highest value in *y* for all coincidences in group 1 (0.30, from coincidence 2).

The top-level node(s) use the supervised mapper instead of the temporal pooler, and so it does not have any groups. The job of the mapper is simply to map coincidences from the spatial pooler to categories obtained from the category sensor file. The mapper assumes that the lower levels have sufficiently discriminated the different categories to create a clean mapping between coincidences and output categories.

### 5.3. Design of HTM for Eating/Drinking Detection

In our application, the data for eating/drinking from accelorometer is quite different from that in the image processing problem which is the typical application of HTM algorithm. In this case, we have to design the HTM framework and define how to use HTM for our experimental data set.

**Figure 4.**
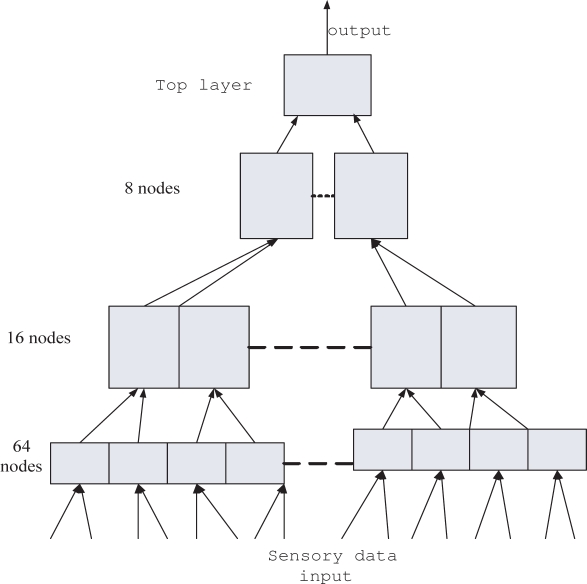
Our design for eating or drinking application.

The HTM applied for eating/drinking identification is designed as a 4-layer structure (Figure 4). We set the sensor data input layer to have 64 input nodes and the sensor data length for one activity is 300. We choose the second layer 16 nodes and the third layer 8 nodes, respectively. The top layer is the classification result layer with one node only.

The input to the HTM is a buffer carrying 256 data sets long with each set consist of 3 values (accelerations in *x*, *y* and *z* directions, while the length of the buffer is time). One such buffer represents a single eating or drinking activity, like bringing a piece of broccoli on a fork to your mouth and putting the fork back down. Table 1 shows the data buffer with each line contents respectively. Then we construct 64 level 1 nodes, each reading 3x4 (256/64=4 data sets for each node and 3 values of *x_i_*, *y_i_*, *z_i_* for each data set) patch of the values. Level 2 combines 4 level 1 nodes and level 3 combines 2 level two nodes, level 4 is one node trained in supervised mode.

During training phase, we scroll the sensor input data from left to right of the data buffer (see Table 1), separating input with all 0 in the separating line when the single activity is done, this means in line 62 in Table 1, we have to input a line with all elements zero. During inference, after the network was fully trained, for better accuracy we scroll the buffer in a similar manner, and sum the solutions for better accuracy.

In order to explain clearly how to use the above data buffer during training phase, see Table 1, we use 256 samples of a total of 316 data (example for one activity), 3 numbers for each sample (*x*, *y*, *z* acceleration) into one line of a text file, so we have 768 numbers total in one line. Because one eating/drinking activity has 316 (even sometimes longer or shorter) of *x*, *y*, *z* samples, we need to repeat this line 61 (316 – 256 + 1 = 61) times, each time removing one sample (3 numbers) at left, and adding the next one at right. The 62th line would also have 768 numbers, but all being 0 (zeroes) to separate them from the next eating sample. This will form the sensor data file using the data buffer.

A corresponding category file for the sensor data file should be set up for the purpose of training. For example, the category for eating activity repeated 61 times, would have 61 lines, each having one number, e.g. 1 meaning eating, and the zeroes line (blanks) should have 0 as category. The next activity should be for example, “drinking”, and it would again have 61 lines as explained above, with corresponding lines in the category file with number 2 meaning “drinking”, again separated with 0 from the next activity.

We give the network many such activities to train on, in our experiment, at least a dozen of each and better hundreds (but it is time consuming).

During the testing phase, we will use the test data that is totally different from the training data (different person’s activities) by repeating the above steps to build up the data buffer. The HTM can output a very accurate result not only for the data that partially belongs to the training data but also for the data that are totally different from the training data.

It is noted that the width of the data buffer for the training data will influence the results of the classification. The best width of the data buffer should be chosen at least one period data samples of the activity for the repetitive or continuous eating/dring. For example, if the repetitive or continuous eating has 1000 measurement data, we can choose 256 or 516 data for each line of the data buffer, however, if 256 data is less than the data in one single eating activity, we have to choose 516 data in order to get better training results.

## 6. Experimental Results

**Figure 5.**
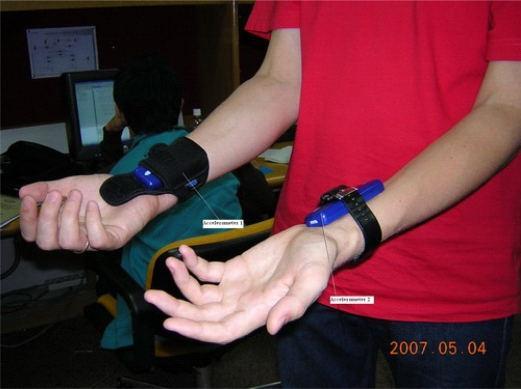
The experiment with 3 axis accelerometer.

**Figure 6.**
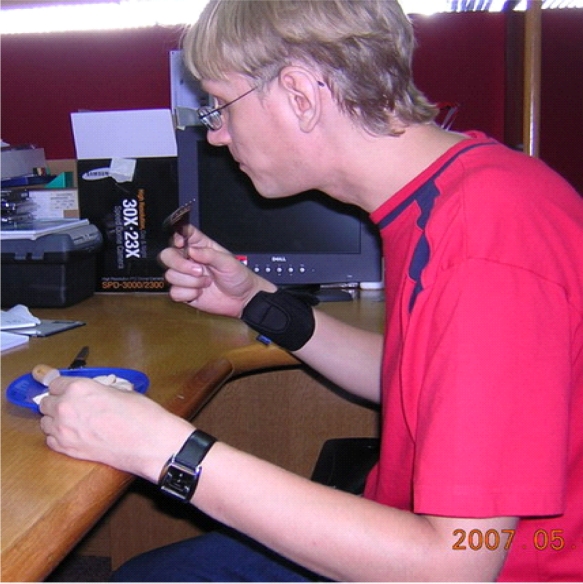
The eating detection experiment.

The experiments are conducted by the system introduced in Section 3 which includes a three axis accelerometer. The accelerometer is attached on both wrists and eating/drinking activities are performed, see Figure 5 and Figure 6. The single eating/drinking activity and continuous eating/drinking activities are both tested in the experiments. The proposed two- stage eating/drinking detection approach is applied to the experimental data. Before the experiments, sensor calibration is done. From the calibration, we confirm that the readings of the accelerometer includes the acceleration due to gravity, i.e., when the wrist does not move, the sensor reading is (0, 0, –*g*).

**Figure 7.**
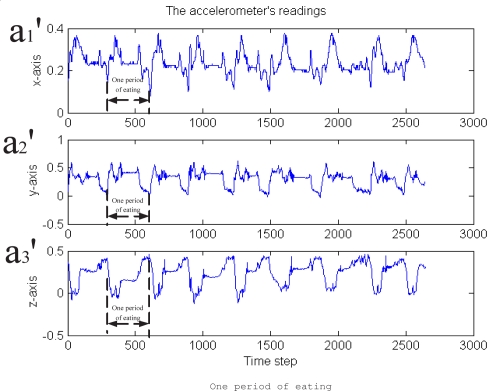
The raw sensor data from the 3 axis accelerometer of eating action.

The first experiment is for eating activity detection. We acted by using the real plate, food and forks and performed single eating activity and continuous eating activity, respectively. The sensor raw data for the continuous eating activity can be seen in Figure 7. The figure gives the three axis acceleration value at each time instant. For single eating activity, the data is one period of the continuous eating data. The second experiment is for drinking activity. Several drinking activities are conducted. The drinking raw sensor data can be seen in Figure 9.

**Figure 8.**
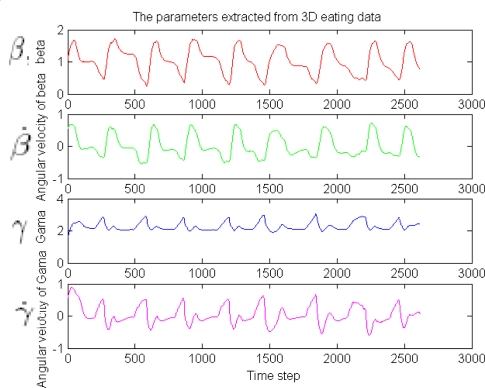
The features extracted from the 3 axis accelerometer of eating activity.

**Figure 9.**
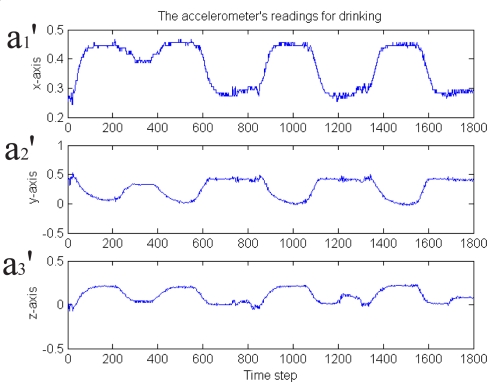
The raw sensor data from the 3 axis accelerometer of drinking activity.

**Figure 10.**
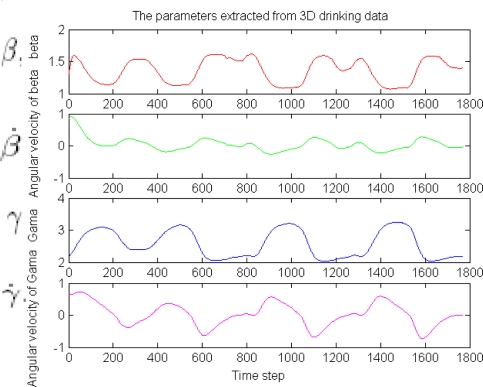
The features extracted from the 3 axis accelerometer of drinking activity.

From these figures, we know that the raw sensor data is very noisy. In order to detect and distinguish the eating and drinking activities more robust and effectively, the feature extraction algorithm proposed in Section 4 is firstly used. The feature extraction results are shown in Figures 8 and 10, respectively. The features extracted in these figures are Euler angles and their angular rate – *β*, *β̇* and *γ*, *γ̇*. Although the angular *α* and *α̇* are not used here because we apply one sensor only (the information is not enough, for the reason, see equation (8)), the four features in the figures are enough for classifying the eating / drinking that is verified by the following experimental results from HTM.

After feature extraction, the HTM is applied for the identification of eating and drinking based on the features extracted from the raw sensor data. In order to train the HTM and test it, the feature data is similarly designed as a data buffer that is explained in Section 5.3. It is noted that the data buffer for feature data is different from the raw data buffer because the data set has 4 data width rather than 3 data. See Table 2. We used the extracted features data buffer of a single eating and drinking activity to train the HTM by repeating the data file 10 times. Then the feature data of the different single eating and drinking activities is classified through the trained HTM. The Monte Carlo runs of 20 times is performed. Results are always 100% accurate for both eating and drinking detection.

The other experimental test is for continuous eating and drinking activity detection with the same trained HTM network as the above test. We also conducted 20 Monte Carlo runs on each continuous activities’ classifications and calculated the average successful rate for the classifications. Table 3 gives the results of 10 different group continuous activities that are obtained from different people at different time.

For the comparison purpose, we also use the HTM network itself to detect the eating and drinking activities (using raw sensor data as the input of the HTM without the first step–feature extraction). In this case, the raw sensor data for single activity is firstly made into the data buffer so as to train the HTM network. Then the raw sensor data of the continuous activities is classified by the trained HTM. The experimental results are listed in Table 4.

From the two tables, we can find that the average successful rate is greatly improved when we apply the feature extraction algorithm compared to the case using the raw sensor data as the input to the HTM.

## 7. Conclusions

This paper presented a novel algorithm which was based on EKF and HTM for eating/drinking detection for human activity monitoring of daily life in wireless environments. The proposed method used a simple hardware structure with wireless accelerometers so that the system was easily set up. If a smaller and less expensive accelerometer was used, such as imote2 [28], the system may be more ambulatory and more affordable. For the proposed algorithm itself, the experimental results show that the new scheme can achieve significant classification results even for the very noisy data. However, there are still many issues remaining for future study. Real time algorithms based on dealing with both time-varying and space varying signals or multi-modality sensor based algorithm are both challenging problems for further investigations. For example, if the person uses the same movement for eating and drinking, the results will be wrong because the HTM will classify it as the same activity in this case. How to tackle this false detection problem and improve the successful rate of the activity detection is a key problem. The multi-modality sensor should be used to obtain more information on the eating/drinking activities so as to improve the successful rate of the activity detection in the future.

## Figures and Tables

**Table 1.** Data Buffer.

Line 1	*x*_1_	*y*_1_	*z*_1_	⋯	*x*_256_	*y*_256_	*z*_256_
Line 2	*x*_2_	*y*_2_	*z*_2_	⋯	*x*_257_	*y*_257_	*z*_257_
Line 3	*x*_3_	*y*_3_	*z*_3_	⋯	*x*_258_	*x*_258_	*x*_258_
⋯	⋯	⋯	⋯	⋯	⋯	⋯	⋯
Line 61	*x*_61_	*y*_61_	*z*_61_	⋯	*x*_316_	*y*_316_	*z*_316_

**Table 2.** Data Buffer of Features.

Line 1	*β*_1_	*β̇*_1_	*γ*_1_	*γ̇*_1_	⋯	*β*_256_	*β̇*_256_	*γ*_256_	*γ̇*_256_
Line 2	*β*_2_	*β̇*_2_	*γ*_2_	*γ̇*_2_	⋯	*β*_257_	*β̇*_257_	*γ*_257_	*γ̇*_257_
Line 3	*β*_3_	*β̇*_3_	*γ*_3_	*γ̇*_3_	⋯	*β*_258_	*β̇*_258_	*γ*_258_	*γ̇*_258_
⋯	⋯	⋯	⋯	⋯	⋯	⋯	⋯	⋯	⋯
Line 61	*β*_61_	*β̇*_61_	*γ*_61_	*γ̇*_61_	⋯	*β*_316_	*β̇*_316_	*γ*_316_	*γ̇*_316_

**Table 3.** The success rate of the eating/drinking detection by the HTM algorithm based on raw sensor data.

**Activities**	**The Success Rate**	**Monte Carlo Runs**
Continuous Eating 1	85.117%	20
Continuous Eating 2	86.354%	20
Continuous Eating 3	84.694%	20
Continuous Eating 4	85.249%	20
Continuous Drinking 1	85.765%	20
Continuous Drinking 2	86.008%	20
Continuous Drinking 3	85.121%	20
Continuous Drinking 4	86.136%	20
Continuous Eating and Drinking	84.370%	20

**Table 4.** The success rate of the eating/drinking detection by the HTM algorithm based on features.

**Activities**	**The Success Rate**	**Monte Carlo Runs**
Continuous Eating 1	87.195%	20
Continuous Eating 2	87.709%	20
Continuous Eating 3	87.034%	20
Continuous Eating 4	88.847%	20
Continuous Drinking 1	87.996%	20
Continuous Drinking 2	88.139%	20
Continuous Drinking 3	87.874%	20
Continuous Drinking 4	88.556%	20
Continuous Eating and Drinking	86.465%	20
